# Usability and feasibility of E-nergEYEze: a blended vision-specific E-health based cognitive behavioral therapy and self-management intervention to reduce fatigue in adults with visual impairment

**DOI:** 10.1186/s12913-023-10193-4

**Published:** 2023-11-16

**Authors:** M.H.J. Veldman, H.P.A. van der Aa, H. Knoop, C. Bode, C.T.J. Hulshof, L. van der Ham, G.H.M.B. van Rens, M.W. Heymans, R.M.A. van Nispen

**Affiliations:** 1 Ophthalmology, Amsterdam UMC, location Vrije Universiteit Amsterdam, De Boelelaan 1117, Amsterdam, The Netherlands; 2 Amsterdam Public Health, Quality of Care, Mental Health, Aging and Later Life, Amsterdam, The Netherlands; 3 Departments of Medical Psychology, Amsterdam Public Health research institute, Amsterdam UMC, location University of Amsterdam, Meibergdreef 9, Amsterdam, The Netherlands; 4 Department of Psychology, Health and Technology, University of Twente, Enschede, The Netherlands; 5 Department of Public and Occupational Health, Coronel Institute of Occupational Health, Amsterdam UMC, University of Amsterdam, Amsterdam, The Netherlands; 6 Vrije Universiteit Amsterdam, Amsterdam, The Netherlands; 7 Department of Epidemiology and Data Science, Amsterdam University Medical Centers Boelelaan, Amsterdam, The Netherlands

**Keywords:** Fatigue, Visual impairment, E-health, Cognitive behavioral therapy, Usability, Feasibility

## Abstract

**Background:**

Over 50% of adults with visual impairment experience severe fatigue. Therefore, we developed a guided E-health intervention based on cognitive behavioral therapy and self-management to reduce fatigue in this population. This pilot study evaluated the usability, feasibility, fidelity and potential effectiveness of E-nergEYEze.

**Methods:**

E-nergEYEze was developed by a design team and customized by conducting a pilot study using an iterative development strategy. The intervention was first tested in a usability study among adults with visual impairment (n = 5). Participants were asked to think-aloud while exploring the intervention features and a semi-structured interview was performed afterwards. Subsequently, the enhanced intervention was tested in a feasibility study. Adults with visual impairment and severe fatigue (n = 10) followed the intervention partially with guidance from a social worker and one-time computer trainer support. Fatigue severity (Checklist Individual Strength), fatigue impact (Modified Fatigue Impact Scale) and cognitive behavioral therapy skills (Competencies of Cognitive Therapy Scale-Self Report) were measured at baseline and at three months follow-up and analyzed with the Wilcoxon signed-rank test. The intervention was evaluated through evaluation forms.

**Results:**

The usability study resulted in adjustments to content and lay-out with regard to optically shortened text sentences, separate pages for information and assignments with one read-aloud audio and an additional descriptive explanation of page content. Digital challenges were overcome with mandatory computer training and e-platform modifications. The feasibility study showed a positive trend in reducing fatigue severity (Z -6.108; P < .001; SD 8.4), impact of fatigue (Z − 4.451; P < .001; SD 11.4) and cognitive behavioral therapy skills (Z -2.278; P = .023; SD 19.3). Participants gave useful feedback regarding accessibility, content and guidance, with an overall positive experience. The intervention was rated with a median score of 8 (range 7–10).

**Conclusion:**

We developed, evaluated and optimized E-nergEYEze by applying a user-centered and iterative approach. E-nergEYEze showed a promising trend to reduce fatigue severity and impact of fatigue and to increase cognitive behavioral therapy skills. The study methods were feasible and the fidelity of the intervention protocol was suitable. Performing a randomized controlled trial is warranted to give insight into whether E-nergEYEze is cost-effective in reducing severe fatigue in adults with visual impairment.

**Trial registration:**

International Clinical Trial Registry Platform: NL7764. Date registered: 28-05-2019.

## Introduction

The global population with vision loss has increased to over three billion people [1]. These people seem to be more vulnerable to suffer from severe fatigue [2, 3], which is described as a mental and physical sensation that is uncontrollable, unpredictable and overwhelming. Causes of fatigue that have been described are experiencing a high cognitive load, a large amount and high intensity of activities, a high effort to establish visual perception, difficulty with light intensity and negative cognitions [4]. It affects various aspects of daily functioning and overall well-being, such as difficulties with maintaining energy, concentrating and processing information [4, 5]. A recent meta-analysis showed that the odds of having severe fatigue were significantly higher in adults with visual impairment than in adults without visual impairment (2.61; 95% CI 1.69 to 4.04). However, the studies used in that meta-analysis often had a different focus and fatigue or ‘vitality’ was measured as a secondary outcome [6]. Although severe fatigue seems to be a major problem in people with vision loss [3], treating fatigue in this population has not yet been recognized as a research priority [6].

There is evidence on effective interventions to treat fatigue in other chronic diseases with cognitive behavioral therapy (CBT) and self-management (SM) [7–13]. Insights suggest that beliefs about fatigue and behavior are related to the persistence of fatigue [13]. Interventions aimed at fatigue perpetuating beliefs and behavior, e.g. low activity level, sleep problems and fatigue catastrophizing, seem to be important and associated with a reduction in mental and physical fatigue [13–15]. Guided E-health interventions based on CBT and SM, tailored to specific symptoms and adapted to specific populations, have proven to be successful in the reduction of fatigue [16–20]. These digital treatment developments are progressing, though this has not yet been explored specifically for people with visual impairment [21].

People with visual impairment are increasingly using technology as a tool for safety, mobility, independence and social access [22–24]. However, it is considered a challenge to develop accessible web content and accomplish user experience satisfaction to achieve required goals effectively and efficiently [24–27]. Therefore, the design of an E-health intervention for this target population has to meet the specific needs and unique requirements of people with visual impairment using technical devices. Web content has to be suitable to technical device features and assistive technology such as screen magnifier or screen reader [24, 26]. In addition, a proactive attitude and openness to computer training from a user perspective can improve usability and increase accessibility of web content [24, 27].

Capitalizing on the lack of evidence-based care regarding reducing fatigue severity using digital technology for people with visual impairment, we developed E-nergEYEze, a blended vision-specific E-health intervention based on cognitive behavioral therapy and self-management. We applied a user-centered and iterative approach to develop, evaluate and optimize E-nergEYEze, which have been recognized as key factors for successful E-health products [28–31]. The current pilot study aims to explore usability, feasibility, fidelity and potential effectiveness to determine whether E-nergEYEze is feasible as a future treatment option in people with visual impairment.

## Methods

### Design

A user-centered and iterative approach was adopted in the development of E-nergEYEze and applied during all stages of this pilot study. A usability study was conducted to evaluate and optimize usability, using qualitative methodology. Subsequently, potential effectiveness, feasibility of study methods and fidelity to the research protocol were examined in a feasibility study, using both qualitative and quantitative methodologies.

### Intervention development

E-nergEYEze was developed by a design team represented by patients with visual impairment (n = 3), social workers (n = 2), psychologists (n = 3), information and communication technology (ICT) trainers (n = 2) of two large low vision service organizations in the Netherlands (i.e. Royal Dutch Visio and Bartiméus), and researchers (n = 5) of Amsterdam University Medical Centers and the University of Twente. They collaborated to develop the content of the intervention based on CBT and SM, tailored to user needs. Themes and content were inspired by the E-health interventions E-PsEYE [32] and Dia-fit [16], practice-based expertise and taking into account perpetuating factors and determinants of fatigue reported by visually impaired persons [3, 33, 34]. All members of the design team equally contributed and iteratively evaluated the content of the intervention. Researchers of the Low Vision research group at Amsterdam UMC finalized the content in a final discussion with psychologists (n = 2) from the low vision service organizations.

Content consisted of nine modules informing the participant on how to cope with fatigue, focusing on vision-specific and fatigue-related beliefs and behaviors. The first module was an introduction on understanding vision-related fatigue and setting personal goals, followed by eight thematic modules on: (1) Dealing with visual impairment and setting personal goals; (2) Formulating helpful fatigue-related beliefs; (3) Graded activity program; (4) Communication and social support; (5) Relaxation; (6) Improving sleep; (7) Work optimization; and (8) The future. The modules were programmed into the E-health platform (e-platform) ‘Minddistrict’ (http://www.minddistrict.com), initially created with all available features available (e.g. colored text areas, fold-out text areas, picture, audio, video, diary). Each module started with a video of a healthcare professional who introduces the theme, motivates and creates recognition, followed by pages with information and assignments. All pages included read aloud audio per paragraph and short stories from fellow visually impaired people. Accessibility within the e-platform was iteratively tested by good-sighted and visually impaired ICT experts on IOS and android using technical device features (voice-over) and assistive technology (i.e., screen reader, screen magnifier and braille translation software). Technical bugs within the e-platform or content inconveniences when using assistive technology were discussed and resolved by the executive researcher or the e-platform.

As described in our protocol paper [35], in the feasibility study, participants were guided by a social worker, starting with one face-to-face session to discuss the purpose and expectations of the intervention, time planning, digital communication and possibilities to ask for support when symptoms worsened. Participants were able to follow E-nergEYEze at home or at any other preferred place on a technical device. An optional computer training was offered with instructions on the e-platform in the web browser and/or app to provide support for less digitally proficient participants. Guidance by the social worker was pursued digitally through message contact within the e-platform after each completed module. Social workers were supervised by psychologists for support on digital communication or to discuss specific cases if needed. All involved social workers (n = 5), computer trainers (n = 4) and psychologists (n = 2) were trained in applying the study procedures, delivering face-to-face and digital guidance, and in using the e-platform in a one-day session.

### Recruitment

**Fig. 1:**
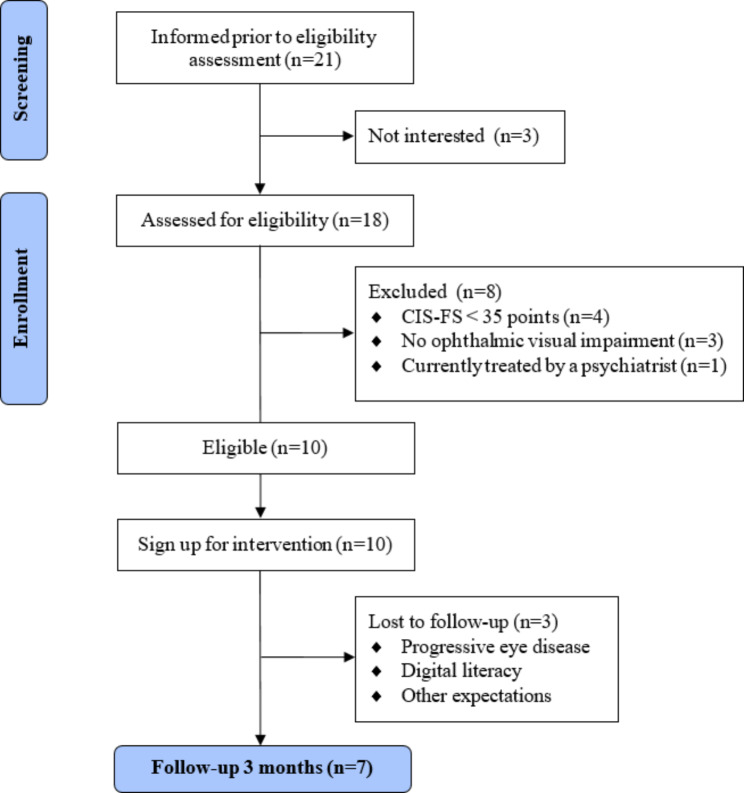
Overview participant flow feasibility study

Participants were recruited through purposive sampling by social workers from the two low vision service organizations in the Netherlands between October 2019 and June 2020. Inclusion criteria for the usability study were: (a) having an ophthalmic visual impairment (visual acuity ≤ 6/18 and/or ≤ 30 degrees concentric visual field), (b) being ≥ 18 years and (c) understanding the Dutch language. For the feasibility study, participants had to meet the additional inclusion criteria of: (d) experiencing severe fatigue (Checklist Individual Strength – subscale Fatigue Severity: CIS-FS ≥ 35 points [36]), and (e) having access to the internet. The exclusion criterion for both studies was (a) experiencing severe cognitive limitations assessed with the 6-item screener (short validated Mini-Mental State Examination) [37]. An additional exclusion criterion for the feasibility study was: (b) currently receiving treatment, or having received treatment in the last 12 months from a medical specialist for a comorbid disease that could be the main cause of fatigue (multiple sclerosis, cancer or a psychiatric disorder). Figure 1 shows an overview of the participant flow of the feasibility study.

### Procedure usability study

Usability was evaluated by five participants with visual impairment (Table 1) [38, 39] at home (n = 4) or at a low vision service organization location (n = 1) using the think-aloud method [40]. Participants were asked to freely explore the intervention on a preferred technical device with assistive technology as needed, without receiving program specific instructions. More specifically, the following script was used: ‘*I would like to ask you to read or have the voice-over read the text that appears on the computer screen and complete the assignments. In addition, I would like to ask you to speak aloud any thoughts you have. Do not be embarrassed to express certain thoughts. We would like to know exactly what is going through your mind; this could be anything.*’ While going through two or three modules, participants vocalized thoughts, feelings and opinions. Afterwards, a semi-structured interview was performed to gather additional qualitative information on the design, content and lay-out. Participants gave their opinion on strengths, limitations and suggestions for improvement. All sessions were audio-recorded with participants’ consent.

### Procedure feasibility study

After improving E-nergEYEze following the results of the usability study, a feasibility study was conducted among ten severely fatigued adults with visual impairment (Table 1). As a result of the COVID-19 pandemic, recruitment was delayed. Therefore, the pilot study was shortened in time (3 instead of 5 months) and participants (10 instead of 12 participants). Participants followed four out of nine modules under guidance, with each module of the intervention being tested by at least one participant. Telephone interviews were conducted at baseline (T0) and after three months (T1) consisting of three questionnaires with a process evaluation at T1. The social workers completed a process evaluation at T1. All responses were entered into Castor (data entry software) [41]. Fidelity was measured with the time spent to complete all modules, the number and nature of logins (web browser or application), and the frequency and nature of contact with the social worker (digitally or by telephone) within the e-platform.

**Table 1:** Participant Characteristics

Characteristics		Usability study (n = 5)	Feasibility study (T0, n = 10)
Gender (male)	N (%)	4 (80)	6 (60)
Age (years)	Mean (SD) Median (range)	54 (17.2) 62 (34–70)	50.3 (12.4) 44 (41–74)
Country of birth (the Netherlands)	N (%)		9 (90)
Eye disease	Retinal disease, N (%)	4 (80)	8 (80)
	Glaucoma, N (%)		1 (10)
	Corneal disease, N (%)	1 (20)	
	Albinism, N (%)		1 (10)
LogMAR (visual acuity best eye)	Mean (SD) Median (range)		0.8 (0.5) 0.8 (0.5-2.0^a^)
Eyesight, subjective	Fair, N (%) Poor, N (%) Very poor, N (%) Blind, N(%)		2 (20) 4 (40) 3 (30) 1 (10)
Employment (yes)	N (%) Mean hours (SD)		4 (40) 26.0 (15.6)
Volunteer work (yes)	N (%) Mean hours (SD)		5 (50) 13.6 (14.9)
Education (years)	Mean (SD) Median (range)		11.1 (1.3) 12 (9–12)
Marital Status	Unmarried, N (%) Married, N (%) Divorced, N (%)		3 (30) 6 (60) 1 (10)
Living alone (yes)	N (%)		4 (40)
Somatic comorbidity	No comorbidity, N (%) 1 comorbid disorder, N (%) ≥ 2 comorbid disorders, N (%)		3 (30) 1 (10) 6 (60)
Fatigue severity	Mean (SD) Severe fatigue (score ≥ 35), N(%)		41.4 (3.0) 10 (100)
Impact of fatigue	Mean (SD)		41.4 (8.7)
Cognitive behavioral therapy skills	Mean (SD)		107.1 (27.0)

^a^including blindness

### Ethics

The pilot study was approved by the Medical Ethics Committee of Amsterdam University Medical Centers, location VU Medical Centre in Amsterdam. All participants gave written informed consent prior to participation and where enrolled by the executive researcher. Taking into account the vulnerabilities and capacities of the target population, the information letter contained a larger font and people could contact the research team via phone or email with questions.

### Outcome measure

The primary outcome was fatigue severity measured with the CIS-FS. Fatigue severity is a subscale of 8 items, ranging from 8 to 57 points with ≥ 35 points indicating severe fatigue and is considered a valid and reliable tool [42]. The secondary outcomes were the Modified Fatigue Impact Scale (MFIS), measuring the impact of fatigue [43] and the Competencies of Cognitive Therapy Scale-Self Report (CCTS-SR), measuring cognitive behavioral therapy (CBT) skills [44]. Higher scores indicate greater impact of fatigue and more mastery of CBT skills. A process evaluation was performed using the Dutch Mental Healthcare thermometer (MHT) questionnaire [45], measuring participant satisfaction, and the Therapist Satisfaction and Adherence, measuring recall and compliance of participants.

### Qualitative analysis usability study

Data from the usability study were qualitatively analyzed using an inductive approach. Relevant information of the recorded files were transcribed, after which data was organized to show and interpret patterns for recurrent themes by the executive researcher [46].

### Statistical analysis feasibility study

Data from the feasibility study were converted from Castor to the statistical software package SPSS for Windows version 26 (SPSS IBM, New York, USA). Quantitative data were analyzed using descriptive statistics and qualitative data were analyzed by the executive researcher using a thematic approach. In addition, user login history data from the e-platform was extracted and analyzed. As a result of the small sample size, quantitative data from primary and secondary outcomes pre-post intervention were analyzed with the Wilcoxon signed-rank test. Multiple imputation (MI) chained equations were applied for missing data post intervention with 20 iterations under the assumption that data were missing at random [47]. A small sample imputation procedure called “midastouch” was used at the item score level [48, 49] to predict missing values for a given variable based on other observed variables according to imputation guidelines [50]. Five imputed datasets were generated and combined into a pooled set using Rubin’s rules. The imputations were carried out in R 4.1.2 [50, 51].

## Results

### Usability study

Most participants (4/5) used assistive technology (e.g. screen reader and/or screen magnifier). Overall, participants would recommend the intervention to others (4/5) and were personally interested in following the intervention (3/5). Thematic analysis of the think aloud method and semi-structured interviews resulted in three main themes.

### Digital challenges

Participants reported a lack of clarity on how to use the e-platform and follow the modules: the meaning of e-platform buttons were unclear (4/5), entering an answer by typing was difficult (4/5), it was difficult to activate the e-platform account at the start (3/5), it was unclear how to navigate through the e-platform (3/5) and the red error message that occurred when an assignment text area was not completed was unclear (1/5). More specifically, it was unclear where to start the training, on which page the participant was on, how to move to the next page, how many pages there were in total per module, how to share answers with the social worker and where to find the diary. Participants also experienced technical bugs, for example the cursor focus was not automatically situated at the top of a new page (3/5), the screen reader skipped unlabeled text (1/5) and diaries could not be opened with the screen reader activated (1/5).

### Lay-out

Participants noted several inconveniencies and obscurities regarding the lay-out. Most importantly, it was difficult to obtain an overview of the overall text (4/5), big pictures had no added value (4/5), text that was put in colored text areas gave insufficient contrast (3/5), read aloud audio had a small play button (3/5), contrast of the entire screen could not be flipped into black-white (2/5), too much information on one pages (2/5), text size was not adjustable (1/5) and fold-out text areas were confusing (1/5).

### Content

Participants gave feedback regarding the module content: it was unclear that read aloud audio and written text contained the same information (4/5) and that there were assignments at the bottom of a page (4/5), it was confusing that there were multiple read aloud audio’s on one page (3/5), it was difficult to follow the instructions in the introduction module on how to use the e-platform (2/5), it was exhausting to read written text (2/5), titles were unclear (2/5), there were too many different things at one page (1/5) and formulating personal goals in the introduction module was difficult (1/5). Strengths mentioned were the presence of read aloud audio with a pleasant voice and pace (4/5), a catching video at the start of every module (3/5), the presence of stories from fellow visually impaired people (3/5), the structure of the intervention (1/5), and the possibility to re-read assignments (1/5).

### Improvements

Based on these results, we made user-specific improvements to E-nergEYEze, presented in Table 2.

**Table 2:** Results qualitative analysis usability study

Theme	Improvements:
Digital challenges	1. Mandatory computer training: - Assistance with registration in the e-platform - Instruction on e-platform features (location buttons and their meaning) - Instruction on how to navigate through the intervention (with assistive technology) and how modules are constructed - Instruction on how to dictate answers to assignments
Lay-out	1. All text sentences were optically shortened to create a better overview 2. Pictures were removed 3. Colored text areas were removed 4. Fold-out text areas were removed 5. Two versions of the intervention were created: - Visual: written text and read aloud audio - Non-visual: only read aloud audio (no written text)
Content	1. Information and assignments on separate pages 2. One read aloud audio at the top of every page 3. Descriptive explanation of the content of each page* 4. Setting personal goals with the social worker during the face-to-face session

*total amount of pages, page number, audio time, introductory text, number of assignments, end of page, topic of the next page, how to proceed

### Feasibility study

The feasibility study was conducted during the COVID-19 pandemic, resulting in digital face-to-face sessions between participants and social workers at the start of the intervention. All participants opted for the visual version of the intervention. One participant initially preferred the non-visual version, but switched after one week to be able to re-read information. Three participants dropped out at the beginning of the study (Fig. 1). The first participant was not digitally proficient and had expected an on-site treatment. The second participant had other expectations after scanning through the module content. The third participant experienced a progression of his visual impairment and had insufficient time and energy to continue. Module 3 was assigned to two participants who dropped out of the study and therefore not evaluated in this pilot study.

### Process evaluation participants – MHT questionnaire

Participants received sufficient information about the intervention (7/7) and on the expected outcome of the intervention (6/7). All participants experienced sufficient expertise, trust and respect from the social workers. They felt the content and guidance of E-nergEYEze was the right approach for their problems or complaints with sufficient progress throughout the intervention (7/7). For example, the intervention gave more control over their problems (6/7), they were more able to do things that were important to them (6/7) and were more able to deal with people and situations as an effect of treatment/guidance where they previously experienced problems with (5/7). The effort to correctly follow the intervention was rated with a median score of 8 (range 2–9). One participant rated her effort very low and reflected that her expectation of the module content was less psychological and more practical information. Overall, E-nergEYEze (treatment and/or guidance) received a median score of 8 (range 7–10) and was unanimously recommended to others. Three themes emerged from the open-ended questions: ‘What do you think needs to be improved to increase this rating?’ and ‘Do you have any further comments about the treatment or guidance?’. As a complement, participant experiences of the modules, assignments and overall intervention of the feasibility study are illustrated in Box 1.

### Accessibility

The participants mentioned difficulties scrolling up and down through a page (2/7), navigating (1/7), with the unchangeable contrast (1/7) and the voice-over not being accessible enough when using the application on a mobile phone (1/7). Positive comments were the optically short sentences (1/7), the presence of videos and read aloud audio (2/7) and the convenient use of the application (2/7).

### Content

Participants reported that the expectation of the module content should be discussed in advance (3/7), as well as the type of assignments (1/7) and estimated time investment (3/7). One participant had hoped for more concrete, practical tools, instead of a main focus on mindset. On the other hand, participants mentioned that the module content was described as clear, easy to grasp and useful. The intervention provided guidance, changed thinking skills and behavior patterns, created more self-awareness and raised awareness of having a positive mindset and energy. Filling in the diary was often forgotten, for which participants would like to receive a daily reminder (3/7).

### Guidance

Participants were positive about the overall guidance (7/7). The social workers were pleasant during contact moments, took time to answer questions and provided feedback with sufficient depth. One of the participants preferred more intense contact with regard to the module content.

**Box 1:** Participant experiences feasibility study

Module	Quotes
Module 1 Dealing with visual impairment	*“I gained insight into on-going processes and recognized pitfalls. I have to learn not to respond impulsively, but to change old patters or behavior. Questions in assignments encourage thinking and self-mapping of problems” (42y, male)* *“It makes me aware of what I am doing and how I can deal with my disability” (52y, male)*
Module 2 Formulating helpful fatigue-related beliefs	*“Especially the module on helpful beliefs made me more aware of what my habits are in daily life. I regularly seek distractions by closing my eyes with headphones on, in this exercise I could find myself well” (44y, female)* *“I applied helping thoughts multiple times and focused on how I could change my mind to a different mindset” (74y, female).*
Module 3 Graded Activity	Not evaluated
Module 4 Communication and social support	“*The participant I guided, worked on her assertiveness and how to deal with the social environment. This module gave her practical tools, especially during the COVID-19 pandemic” (according to the social worker guiding a 74-year-old female).*
Module 5 Relaxation	*“After a working day, the relaxation exercises made me conscious about relaxing and consciously closing my eyes gave them a rest” (44y, male)*.
Module 6 Improving sleep	*“I slept badly, now I know where it comes from. The module about sleep gives more support, I am able to find peace now” (44y, female)*
Module 7 Work optimization	*“I have to divide activities into parts of the day or several days, I want and do too much and need to start making choices” (42y, female)*
Assignments	“*Writing down and reading back your answers is helpful to analyze yourself. The questions encourage you to think and map out problems.” (42y, male)* *“It is helpful to answer open-ended questions” (44y male)*
Diary	*“Personally, I find it a big task to fill out diaries structurally, although I understand very well that it is a substantial part of the process” (52y, male)*
Overall experience	*“The combination of getting started at home and sending in answers for feedback is a good match. I am able to do new things, which I would not be able to do before. This training is enriches my process of dealing with a visually impairment” (42y, male).* “*I started thinking about things more consciously. I became aware of the fact that it is allowed to be sad and cry. I am able to explain my fatigue better to my social environment and I am better able to do things outside and take rest as well. The visual impairment is part of me, but I am part of our society too.” (female, 44).* *“Surprisingly, the training made a few things clear in established patterns and strategies. I found the training very rewarding to follow” (74y, female)*

### E-platform user login history

The seven participants who followed E-nergEYEze spent a median of 11.5 weeks (range 6–15) on completing four modules. One participant exceeded the time period of three months and explained that she wanted to do really well in the module on work participation that took a little longer. The number of times logged into the e-platform was median 16 (range 9–32), of which median 11 times via the web browser (range 3–18) and median 9 times via the app (range 0–30). Five participants used both platform services and two participants only used the web browser. The face-to-face sessions took a median time of 90 minutes (range 45–120) and the number of contact moments between participant and social worker was median 6 times (range 3–11), of which 85% by message contact and 15% by telephone. The social worker spent median 120 minutes (range 65–210) guiding a participant in the e-platform.

### Therapist process evaluation

All social workers (5/5) were satisfied with the face-to-face sessions and according to them most participants (4/7) gave sufficiently detailed answers to the assignments. Three participants gave answers that varied in comprehensiveness or were insufficient. The digital aspect of providing written feedback went well for most social workers (3/5), while some found it a positive challenge (2/5). Illustrative examples are: *“It was my first time working with clients digitally and I had to practice observing what was being written and carefully respond” (female, 57y)* and *“It takes time to think carefully about which words to use and how they come across or are perceived by the participant” (female, 48y).* Social workers positively rated the structure of the intervention with clear homework assignments (4/5). They remarked: *“The modules provide a clear description of problems with a theoretical foundation, I think many people with a visual impairment recognize these problems.” (male, 62y)* and *“Writing down answers is a process, putting thought into words and in this way learning to deal with fatigue.” (female, 46y).* Three social workers found it difficult to obtain an overview of the e-platform with regard to providing feedback.

### Potential effectiveness

The Wilcoxon signed-rank test showed a positive trend post versus pre-intervention. We measured a reduction of fatigue severity (p < .001), fatigue impact (p < .001), and an increase of CBT skills (p = .023). Fatigue severity was reduced by 12.2 points on the CIS-FS ( > = 35 points indicating severe fatigue), the average score regarding the impact of fatigue was reduced by 9 points and CBT skills increased with an average score of 6 points (Table 3).

**Table 3:** Before and after intervention measures feasibility study N = 10 (after multiple impution)

Outcome measures	T0	T1	z-score	P value
	Mean (SD)	Mean (SD)		
Checklist Individual Strength, subscale Fatigue Severity^a^	41.4 (2.9)	29.2 (8.4)	-6.108	< 0.001
Modified Fatigue Impact Scale^a^	41.4 (8.7)	32.0 (11.4)	-4.451	< 0.001
Cognitive Therapy Skills - Self Report^b^	107.1 (27.0)	113.4 (19.3)	-2.278	0.023

^a^Lower score indicating less fatigue

^b^higher score indicating more mastery of CBT skills

## Discussion

The main focus of this pilot study was to evaluate and optimize E-nergEYEze, an E-health intervention to reduce fatigue in adults with visual impairment. The usability study resulted in adjustments based on participants’ feedback on the content and lay-out of E-nergEYEze. Digital challenges for people with visual impairment were identified and largely remedied. The feasibility study showed a positive trend in reducing fatigue severity, impact of fatigue and cognitive behavioral therapy skills. Participants’ experience was overall positive and E-nergEYEze was rated with a median score of 8 (range 7–10).

We want to emphasize on the importance of conducting a pilot study of an E-health intervention before further testing or implementation. E-health is on the rise, but conducting pilot studies for successful implementation lags behind [28]. Usability of E-health is very challenging, especially for people with disabilities, and needs to be specified to achieve user satisfaction [26–29]. Therefore, a user-centered and iterative approach are described as key factors in the development of E-health for people with visual impairment [24] and for people who experience severe fatigue [29]. This approach contributed significantly in both the development and evaluation of our intervention. Our pilot study was conducted in a heterogeneous group of participants with vision loss of different severity and caused by various eye diseases. This way, we ensured that as many user requirements as possible are aligned with user needs, preferences and behaviors. Our results are supported by similar research, where the usability and feasibilty of E-PsEYE was tested, an E-health intervention to reduce anxiety and depression in patients with retinal exudative diseases [52].

The usability study provided detailed data where researcher judgement was necessary to determine reflective themes [53]. Across the three themes that emerged (digital challenges, lay-out and content), most feedback was related to navigating, completing tasks, contrast and gaining overview. Specific improvements were made based on these results (Table 2). Technical bugs within the e-platform were discussed with the e-platform company, who were willing to make changes in short order. Even though assistive technology, such as screen magnifier and screen reader, have removed many barriers for this target population [54], it would have been helpful if we could have identified whether feedback related to accessibility was a result of digital proficiency of the user or accessibility of the online platform [55].

The feasibility study has been conducted in a pragmatic design to reflect real-world settings and enhance external validity and generalizability [56]. Beforehand, it is important to clearly discuss expectations of an E-health intervention in a study among people with visual impairment. This could be improved by verbally explaining the digital aspect and expected content of the intervention. During the pilot study, improvements based on the results of the usability study were perceived as positive during the feasibility study; among other things the adaptation to shortened text sentences. We believe the mandatory computer training gave participants in the feasibility study the necessary tools and confidence to work in the e-platform and to ask for help if needed [27]. The non-visual version (only audio), designed to reduce the effort for visual perception [4] and encourage participants to close their eyes, was not utilized. Social workers felt the digital face-to-face session to be satisfactory. The time spend to guide a participant (median 120 min) is relevant for scheduling and budgeting of social workers for future research and potential implementation. Using an e-platform and providing digital feedback was considered a challenge to some social workers. As more experience is gained, we expect that it will become easier and will take less time to provide feedback [57]. The quantitative results of the feasibility study on potential effectiveness were very promising. Even though we used nonparametric tests because of the small group of participants in this pilot study, our results should be interpreted with caution due to the following reasons. An important aspect of fatigue reduction presented in module 3 ‘Graded activity program’ was not evaluated because these participants dropped out early [14, 58]. Still, the relevance of statistical results could also be limited because the intervention was under development and participants followed only part of the intervention. Although, at the moment, MI at item score level is the best way of dealing with missing data for this pilot dataset to increase accuracy of the regression model estimates, it could have resulted in a slight over- or underestimation of the outcomes especially due to the small group [49].

Evidence to support the use of E-health for people with visual impairment is limited [21], but E-health offers advantages with respect to independence in time and place, no transportation difficulties and the stimulation of patient empowerment [59] Our E-health intervention capitalizes on the principle that innovative technology provides opportunities for people living with visual impairment to overcome challenges encountered in their everyday lives [24]. All the specific components of E-nergEYEze were investigated and we think our study provides important information on the usability and feasibility of the complete intervention. We would recommend to address all relevant themes developed to reduce the severity of fatigue in the intended treatment period of five months in future research. It would be valuable to explore whether E-nergEYEze is cost-effective for participants with visual impairment and severe fatigue, as well as predictors and mediators regarding the outcome and for which patients E-health is a suitable treatment option.

## Conclusion

E-nergEYEze was developed with patients and professionals to ensure an E-health intervention tailored to the needs of adults with visual impairment. As a result of this pilot study, the intervention seems to be usable and feasible, and has adequate fidelity. The intervention is potentially effective with respect to the reduction of fatigue and an increase in CBT skills. Our next step is to investigate E-nergEYEze in a randomized controlled trial.

## Data Availability

The datasets used and/or analyzed during the current study are available from the corresponding author on reasonable request.

## Abbreviations

CBT: Cognitive Behavioral Therapy
SM: Self-Management
ICT: Information and Communication Technology
e-platform: E-health platform
CIS-FS: Checklist Individual Strength – subscale Fatigue Severity
COVID-19: Corona Virus Disease 2019 caused by SARS-CoV-2
T0: Baseline assessment
CCTS-SR: Competencies of Cognitive Therapy Scale – Self Report
PIC: Punctate Inner Choroidopathy
RP: Retinis Pigmentosa
